# Is a combined proximal femoral osteotomy necessary when performing open reduction and Dega acetabuloplasty in walking-age children with developmental dysplasia of the hip

**DOI:** 10.1051/sicotj/2026031

**Published:** 2026-06-11

**Authors:** Jinghang Yu, Lianyong Li, Liwei Shi, Lijun Zhang, Qiwei Li, Enbo Wang

**Affiliations:** Department of Pediatric Orthopedics, Shengjing Hospital of China Medical University Shenyang City PR China

**Keywords:** Femoral osteotomy, Walking-age, Children, Developmental dysplasia of the hip, Avascular necrosis

## Abstract

*Purpose:* To determine the necessity of simultaneous proximal femoral osteotomy (PFO) during open reduction (OR) and Dega acetabuloplasty (DA) in patients with developmental dysplasia of the hip (DDH) by comparing the clinical and radiographic outcomes of DDH patients who underwent OR and DA with or without PFO. *Methods:* We retrospectively analyzed 61 DDH patients (72 hips) who underwent OR and DA at our hospital between January 2011 and December 2015. Finally, 52 patients (59 hips) with Tönnis types I, II, and III dislocation were included. We included 28 patients (31 hips) with a median operative age of 19.0 (17.0, 25.0) months (range, 15–36 months) in the PFO group. We included 24 patients (28 hips) with a median operative age of 16.0 (16.0, 23.5) months (range, 14–32 months) in the non-PFO group. Hip joint development was compared by measuring the following indicators: acetabular index (AI), femoral epiphyseal height-to-width index (HWI), et al. In both groups, we calculated changes in AI up to the final follow-up, namely ΔAI (postoperative – final follow-up). *Results:* Mean follow-up durations of the PFO and non-PFO groups were 42 months (range, 24–70 months). ΔAI (postoperative – final follow-up) was 5.6° (range, −15° to 24°; SD 9.0°) and 4.7° (range, −3° to 13°; SD 4.6°), respectively (*t* = −0.492, *P* = 0.625). HWI in both groups at the final follow-up was 55.9 (range, 43–76; SD 7.1) % and 53.1 (range, 43–68; SD 6.1) %, respectively (*t* = −1.654, *P* = 0.104). *Conclusion:* PFO is unnecessary when OR and DA is performed in walking-age children with Tönnis type III DDH or below. *Level of evidence*: level III.

## Introduction

Developmental dysplasia of the hip (DDH) is a common pediatric orthopedic condition that manifests as acetabular dysplasia and abnormal femoral head coverage. Treatment regimen selection is mainly influenced by the patient’s age and disease severity. Most patients aged 18 months and below can be treated conservatively, while the majority of patients aged >18 months with severe pathological changes require surgical reduction or acetabuloplasty and/or proximal femoral osteotomy (PFO) [1–4].

PFO involves femoral shortening, derotation, and varus osteotomy. Relaxation of soft tissues around the hip joint after femoral shortening osteotomy is beneficial for intraoperative reduction and simultaneous reduction of pressure between the acetabulum and femoral head, which ultimately reduces the risk of avascular necrosis (AVN) [5, 6]. Derotation and varus osteotomy can increase post-reduction femoral head stability and promote correction of the pathological morphology of the proximal femur.

For children who have a high displacement, like Tönnis grade IV DDH and excessive femoral anteversion, a femoral shortening osteotomy is usually required. However, there is considerable controversy regarding the indications for PFO in patients with Tönnis grade III DDH or below. Most researchers consider the pathological increase in femoral neck anteversion (FNA) in DDH patients a secondary change following dislocation, with FNA tending toward a physiological decline. Femoral shortening osteotomy at the dislocated hip joint aims at reducing the pressure between the femoral head and acetabulum. However, in younger pediatric DDH patients with a shorter walking experience, hip joint reduction can be readily achieved, and pressure at the femoral head-acetabulum interface is relatively smaller. Currently, the decision to perform PFO relies more on subjective (surgeon’s experience) than on objective evidence (dislocation severity and FNA magnitude). Therefore, we aim to determine the necessity of simultaneous PFO during open reduction (OR) and Dega acetabuloplasty (DA) by comparing the clinical and radiographic outcomes of DDH patients who had undergone OR and DA with or without PFO.

## Materials and methods

### Clinical data

This study was approved by the medical ethics committee of our institution (approval no.: 2016PS085K), and informed consent was obtained from the participants’ parents or guardians prior to study commencement. We retrospectively collected and analyzed data of DDH patients who underwent OR and DA at our hospital from January 2011 to December 2015. The inclusion criteria were as follows: (1) Patients with simple DDH who underwent one-stage OR and DA with or without PFO; (2) Walking-age children with Tönnis type III DDH or below; (3) Patients who were followed up for more than 2 years; and (4) DDH patients who had not been treated previously. We excluded patients with neuromuscular or syndromic DDH and those with incomplete imaging data.

Finally, 52 DDH patients (59 hips) with Tönnis types I, II, and III dislocation were included. Patients were divided into two groups according to the surgical procedure: PFO (OR and DA with PFO) and non-PFO (OR and DA without PFO). The PFO group included 28 patients (2 boys and 26 girls, 31 hips) with a median operative age of 19.0 (17.0, 25.0) months (range, 15–36 months); the left hip was affected in 11 patients, the right hip in 14 patients, and bilateral hips in 3 patients. The non-PFO group included 24 patients (5 boys and 19 girls, 28 hips) with a median operative age of 16.0 (16.0, 23.5) months (range, 14–32 months); the left hip was affected in 10 patients, the right hip in 10 patients, and bilateral hips in 4 patients.

All patients in the PFO group underwent femoral shortening and derotation, which led to restoration of the femoral anteversion angle to 10°–15° and average shortening of 12.3 mm (range, 8.0–20.0 mm). No patients underwent varus osteotomy.

### Measurement and evaluation of imaging parameters

All imaging parameters were measured and evaluated using the picture archiving and communication system (Neusoft, Shenyang City, China). On the anteroposterior (AP) pelvic radiograph, the degree of dislocation was classified according to the Tönnis method [7], and the acetabular index (AI) and articulo-trochanteric distance (ATD) were measured. The center-head distance discrepancy (CHDD) in unilateral DDH patients was measured using the method by Chen et al. [8]. FNA was measured using three-dimensional computed tomography (3D CT) [9]. Skeletal leg length (SLL) was measured on a full-length standing AP radiograph of both lower extremities. Changes in AI and CHDD up to the final follow-up, ΔAI (postoperative – final follow-up) and ΔCHDD (final follow-up – postoperative) were calculated for both groups. The SLL difference between the lower extremities (affected side vs healthy side) was calculated in unilateral DDH patients at the final follow-up to determine limb length discrepancy (LLD). Other measured parameters included the following:

*Alsberg angle:* This was defined as the angle between the axis of the femoral shaft and a line joining the innermost and the outermost ends of the proximal femoral physis on an AP pelvic radiograph [10].

*Femoral head transverse diameter ratio (r value)*: On the AP pelvic radiograph of unilateral DDH patients, the innermost and outermost ends of the capital femoral epiphysis were identified, and the maximum transverse diameter of the capital femoral epiphysis was defined as the straight-line distance between both ends. The *r*-value was defined as the ratio of the maximum transverse diameter of the dislocated side to that of the normal side. The number and proportion of hips with r values ≤1.15 and >1.15 were calculated for both groups.

**Figure 1:**
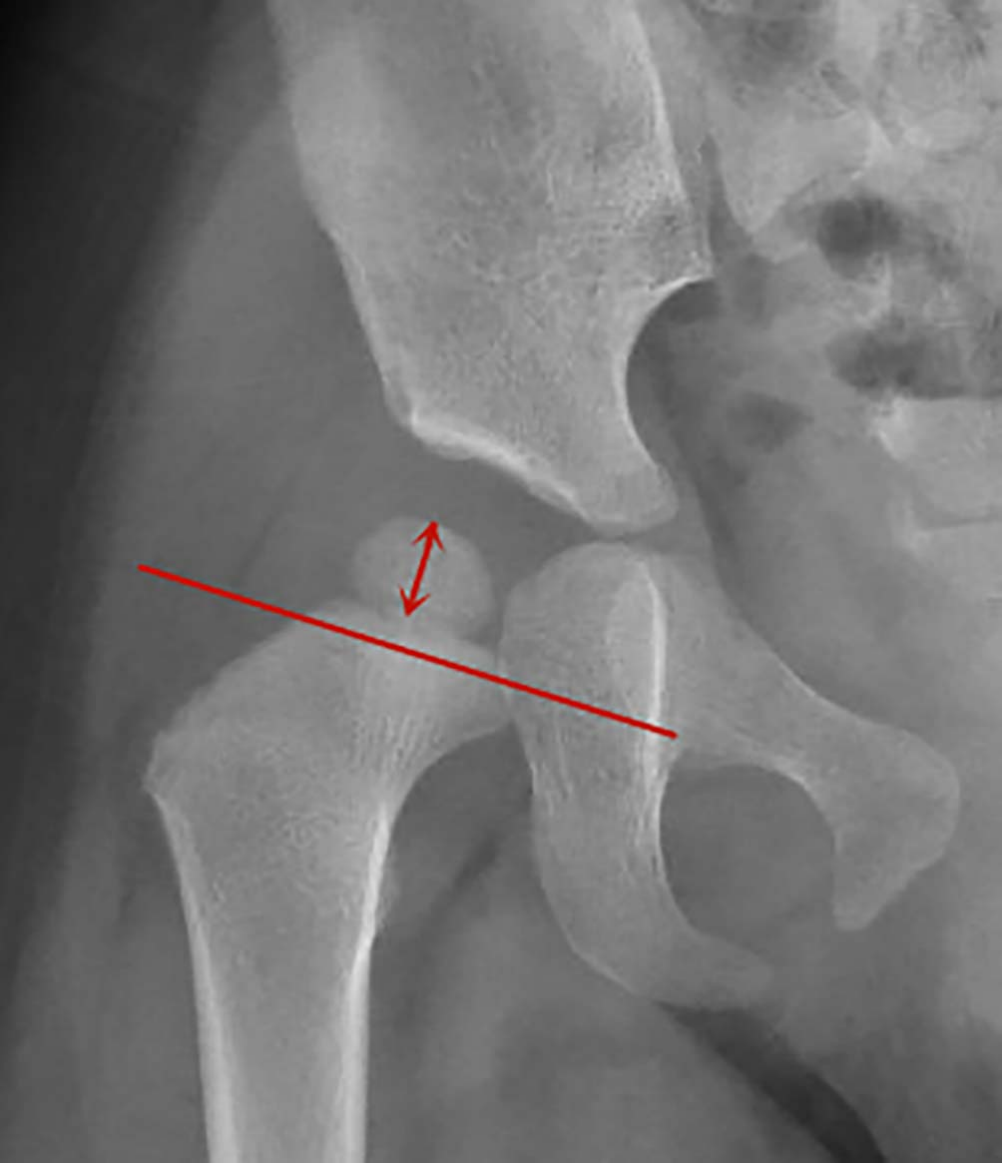
Measurement of HWI.

*The height-to-width index (HWI) of the capital femoral epiphysis*: On the AP pelvic radiograph, a line perpendicular to the direction of the proximal femoral physis was drawn, and the height of the capital femoral epiphysis was defined as the maximum distance of its intersection with the capital femoral epiphysis. HWI was defined as the ratio of the height of the capital femoral epiphysis to the maximum transverse diameter [11] (Figure 1).

Hip joint morphology at the final follow-up was rated using the Severin radiological classification system, and the excellent (Class I)/good (Class II) outcome rate was calculated for both groups. The presence/absence and type of AVN were determined using the Salter criteria and Kalamchi–MacEwen’s classification method, respectively. The proportion of patients with hip joint re-dislocation was calculated for both groups, and clinical outcomes were evaluated using McKay’s clinical evaluation criteria.

### Statistical methods

Statistical analysis was performed using SPSS 24.0 (IBM, New York, USA). The Kolmogorov–Smirnov test was used to test normality. Intergroup differences in preoperative FNA, preoperative AI, postoperative AI, ΔAI (postoperative – final follow-up), Alsberg angle at the final follow-up, LLD in unilateral DDH at the final follow-up, HWI at the final follow-up, operative age, AI at the final follow-up, ATD at the final follow-up, follow-up time, ΔCHDD of unilateral DDH (final follow-up – postoperative), and *r* value of unilateral DDH at the final follow-up were compared using the independent samples *t*-test or Mann–Whitney *U* test. Intergroup differences in proportion of hips with Tönnis types I, II, and III, sex, affected side, distribution of r values of unilateral DDH, proportion of hips with excellent/good Severin and McKay’s scores, and incidence of femoral head AVN were compared using the *χ*^2^-test or Fisher’s exact test. *P* < 0.05 was considered statistically significant.

## Results

DDH classification as Tönnis types I, II, and III did not significantly differ between the PFO group (0, 29, and 2, respectively) and the non-PFO group (1, 26, and 1, respectively) (*χ*^2^ = 1.737, *P* = 0.420). As shown in Table 1, sex and affected side did not differ between groups (*P* > 0.05). The follow-up times in the PFO and non-PFO groups were 43.0 (35.0, 53.0) and 37.0 (29.0, 46.0) months, respectively, which were not statistically different (*Z* = −1.899, *P* = 0.058). The operative age was 19.0 (17.0, 25.0) and 16.0 (16.0, 23.5) months in the PFO and non-PFO groups, respectively, and did not statistically differ (*Z* = −1.691, *P* = 0.091).

**Table 1:** Baseline characteristics of the cohorts, month, M (Q1; Q3).

Parameter	PFO group	Non-PFO group	χ^2^/*Z* value	*P*-value
No. of cases (hips)	28 (31)	24 (28)	–	–
Sex (male/female)	2/26	5/19	1.070	0.301
Affected side (left/right/bilateral)	11/14/3	10/10/4	0.553	0.759
Tönnis types (I/II/III)	0/29/2	1/26/1	1.737	0.420
Follow-up time (month)	43.0 (35.0, 53.0)	37.0 (29.0, 46.0)	−1.899*	0.058
Operative age (month)	19.0 (17.0, 25.0)	16.0 (16.0, 23.5)	−1.691*	0.091

Note: sex, affected side, and Tönnis types were compared using the *χ*^2^ test; follow-up time and operative age were compared using the Mann–Whitney *U* test; *: *Z* value.

The preoperative FNA for the PFO and non-PFO groups were 52.2° (range, 25° to 69°; SD 10.1°) and 50.8° (range, 21°–74°; SD 11.9°), respectively (*t* = −0.503, *P* = 0.617). As seen in Table 2, there was no statistically significant difference (*t* = 0.241, *P* = 0.810) in the preoperative AI between the two groups. The postoperative and final follow-up AI values of the two groups were significantly different (*P* < 0.05), but the difference in the ΔAI at the final follow-up did not significantly differ (*t* = −0.492, *P* = 0.625).

**Table 2:** Comparisons of preoperative, postoperative, final follow-up AI, and ΔAI of the PFO and non-PFO groups, ° Mean, (range; SD), M (Q1; Q3).

Group	No. of hips	Preoperative AI	Postoperative AI	Final follow-up AI	ΔAI (postoperative – final follow-up)
PFO group	31	37.7 (22–50; 6.4)	20.0 (7–39; 8.2)	13.0 (8.0; 20.0)	5.6 (−15 – 24; 9.0)
Non-PFO group	28	38.0 (29–51; 6.0)	27.9 (10–42; 7.5)	24.5 (20.3; 27.8)	4.7 (−3 – 13; 4.6)
*t*/*Z* value		0.241	3.851	−4.012*	−0.492
*P* value		0.810	<0.001	<0.001	0.625

Note: AI: acetabular index; preoperative AI, postoperative AI, and ΔAI were compared using the independent samples *t*-test; final follow-up AI was compared using the Mann–Whitney *U* test; *: *Z* value.

As shown in Table 3, differences in the ATD, Alsberg angle, and HWI at the final follow-up were not statistically significant (*P* > 0.05).

**Table 3:** Comparisons of parameters of the PFO and non-PFO groups at the final follow-up, Mean, (range; SD), M (Q1; Q3).

Group	No. of cases	ATD (mm)	Alsberg angle (°)	HWI (%)
PFO group	31	23.4 (19.8; 27.2)	77.0 (61–90; 8.3)	55.9 (43–76; 7.1)
Non-PFO group	28	25.4 (23.2; 28.2)	76.5 (63–94; 6.9)	53.1 (43–68; 6.1)
*t*/*Z* value		−1.769*	−0.269	−1.654
*P* value		0.077	0.789	0.104

Note: ATD: articulo-trochanteric distance; HWI: height-to-width index; final follow-up ATD was compared using the Mann–Whitney *U* test; final follow-up Alsberg angle and HWI were compared using the independent samples *t*-test; *: *Z* value.

Twenty five patients in the PFO group and 20 in the non-PFO group had unilateral DDH; the LLD at the final follow-up in these patients was 5.2 (2.2, 8.3) mm and 9.6 (6.4, 13.1) mm, respectively (*Z* = −2.855, *P* = 0.004). The difference in SLL was more significant in patients who did not undergo PFO. In both groups, the final follow-up CHDD was larger than the postoperative CHDD. ΔCHDD (final follow-up – postoperative) was 7.0 (range, −7 to 23; SD 8.0) % and 3.2 (range, −12 to 18; SD 7.3) % in the PFO and non-PFO groups, respectively, with the difference being statistically insignificant (*t* = −1.668, *P* = 0.103).

The final follow-up *r* values in unilateral DDH patients were 1.13 (range, 0.79–1.33; SD 0.12) and 1.18 (range, 0.76–1.42; SD 0.15) for the PFO and non-PFO groups, respectively, with the difference being statistically insignificant (*t* = 1.293, *P* = 0.203). In the PFO group, 13 patients (52.0%) had an *r* value ≤1.15, and 12 (48.0%) had an *r* value >1.15; the corresponding numbers in the non-PFO group were 6 (30.0%) and 14 (70.0%) patients. The distribution of r values was not significantly different between the groups (*χ*^2^ = 2.204, *P* = 0.138).

Using the Severin classification system, 4, 21, and 6 hips in the PFO group were classified as Class I, II, and III, respectively, giving an excellent/good outcome rate of 80.6% (25/31). The corresponding numbers for the non-PFO group were 7, 10, and 11, giving an excellent/good outcome rate of 60.7% (17/28), which was not significantly different between the groups (*P* = 0.091).

Based on McKay’s criteria for the clinical evaluation of hip joint function, 1, 13, and 17 hips in the PFO group were rated as acceptable, good, and excellent, respectively, giving an excellent/good outcome rate of 96.8% (30/31). The corresponding numbers for the non-PFO group were 1, 5, and 22, respectively, giving an excellent/good outcome rate of 96.4% (27/28), which was not significantly different from that of the PFO group (*P* = 1.000).

None of the patients suffered re-dislocation during the follow-up. According to the Kalamchi–MacEwen classification, the PFO group had 8 hips with type II AVN, and 1 with type IV AVN, and the non-PFO group had 6 with type II AVN. The incidences of AVN in the two groups were 29.0% and 21.4%, respectively, which were statistically insignificant (*χ*^2^ = 0.449, *P* = 0.503).

## Discussion

In DDH patients aged above 18 months, OR is often required for concentric reduction of the femoral head within the acetabulum. Although the age range of 0–4 years represents the key period for acetabular development, residual acetabular dysplasia is common. Therefore, when correcting acetabular dysplasia, some researchers advocate for simultaneous pelvic osteotomy and OR in children of walking age. Commonly used pelvic osteotomy procedures include Salter, Pemberton, and Dega osteotomy.

Certain researchers have suggested the necessity of simultaneous femoral shortening and derotation osteotomy, a technique first proposed by Ombredanne in 1923, during pelvic osteotomy. Currently, PFO is commonly used with OR and pelvic osteotomy to treat DDH. Tezeren et al. [12] found that the incidence of AVN in DDH patients aged between 3 and 5 years treated with OR and Salter pelvic osteotomy without femoral osteotomy was slightly higher than in those who had undergone femoral osteotomy. Besides, Charki et al. [13] found that clinical outcomes were better and the risk of AVN decreased significantly when a femoral osteotomy was performed, and recommended a femoral shortening osteotomy for high dislocations (Tönnis 3 or 4) in children over 18 months. In addition, Köroğlu et al. [14] reported radical reduction (open reduction and Salter innominate osteotomy combined with femoral osteotomy) yields better clinical and radiographical results in children with DDH undergoing surgery before the age of 4 years. Moreover, Zein et al. [15] reported that one-stage procedure entailing open reduction, Dega pelvic osteotomy, and femoral osteotomy when needed for managing DDH in patients younger than 8 years old revealed acceptable clinical and radiological outcomes; however, there was a higher need for a concomitant femoral osteotomy in patients older than 2.5 years.

However, there are varying opinions on the necessity of simultaneous PFO with OR and pelvic osteotomy. Mootha et al. [16] reported that none of the 12 hips in their study required internal rotation for stability, and no dislocations occurred until final follow-up. Based on these findings, they recommend that femoral derotation osteotomy is not needed in DDH of the early walking age group. Additionally, Wenger [17] recommended that surgeons avoid too great a focus on bony osteotomies, as the management of soft-tissue abnormalities is critical to achieve a stable reduction. Alassaf [18] showed in his study of 435 patients, that femoral shortening did not affect the occurrence of AVN. Furthermore, Duan et al. [19] found that femoral shortening was not required for children aged 2–3 years with Tönnis grade III DDH. What’s more, Kothari et al. [20] indicated open reduction with concomitant pelvic osteotomy was the most appropriate option to provide durable results with the lowest risk of AVN and best radiological and clinical results. In addition, femoral osteotomy increases the risk of bleeding and trauma, resulting in higher economic cost [19], and may also lead to complications such as AVN, postoperative re-dislocation, non-union of osteotomy ends, refracture after removal of internal fixation [21], changes in femoral neck eccentricity, deeper infections [18].

Few studies have evaluated the postoperative outcomes of femoral osteotomy versus non-osteotomy procedures, and most studies lack consistency in intergroup comparisons. Spence et al. [22] compared 38 patients who underwent OR combined with femoral osteotomy with 33 patients who underwent OR combined with pelvic osteotomy and found that the latter procedure was more effective for reversing acetabular dysplasia and maintaining hip stability. In contrast, Kothari et al. [20] reviewed studies of walking-age patients treated either with an open reduction alone or combined with pelvic and/or femoral osteotomies, and indicated there was no evidence that the addition of a femoral osteotomy provided any additional benefit to the patient, although it may be necessary to achieve reduction. However, the groups compared in the aforementioned studies were subjected to different treatment methods. In this study, patients who underwent OR and DA were included as research subjects, and we found no significant differences in parameters, including sex, affected side, follow-up time, operative age, and preoperative FNA and AI values that reflected pathological changes of DDH. Therefore, the treatment outcomes of the two groups were highly comparable.

PFO aims to increase post-reduction hip joint stability, reduce the incidence of AVN, and restore the morphological development of the proximal femur. In the present study, we compared the two treatment groups in the aspects described below.

Post-reduction hip joint stability: Postoperative re-dislocation did not occur in either group. In the non-PFO group, the mean preoperative FNA was as high as 50.8° (range, 21°–74°; SD 11.9°), yet no higher re-dislocation rate was observed in the final follow-up, which may be attributed to the DA increasing anterior coverage and compensating for the high FNA. In addition, most researchers consider the pathological increase in FNA in DDH patients a secondary change following dislocation, with FNA tending toward a physiological decline. Therefore, PFO did not affect the post-reduction stability. We also evaluated the outward displacement of the hip joint center during postoperative development. CHDD is a reliable indicator used to measure the reduction of the femoral head center and is also significantly related to the prognosis. Chen et al. [8] showed that CHDD >6% indicated a poor prognosis. In the present study, unilateral DDH patients in both groups exhibited a slight increase in CHDD after postoperative follow-up for at least 2 years, which did not significantly differ between the two groups. Therefore, our results have confirmed that performing PFO simultaneously during OR and DA did not affect the hip joint stability.

Morphological development of the proximal femur: There were no significant differences in the ATD and Alsberg angle between the two groups. Consequently, whether PFO was performed did not significantly affect the femoral neck growth, orientation of the proximal femoral physis, and other aspects of morphological development.

Morphological development of the hip joint: To eliminate the effects of differences in the criteria for AI correction among different surgeons, we evaluated the changes in parameters related to acetabular development between the postoperative period and the final follow-up. ΔAI did not significantly differ between the two groups, which indicates that PFO did not affect the development and remolding ability of the acetabulum. The difference in the excellent/good Severin radiographic outcome rates between the two groups was statistically insignificant, which also supports the conclusion stated above. Both groups also showed similar excellent/good outcome rates based on McKay’s hip joint function evaluation criteria. Therefore, hip joint function was not affected by PFO.

AVN incidence: The PFO and non-PFO groups had similar AVN incidence rates of 29.0% and 21.4%, respectively. Although all cases in this study were followed up for at least 2 years, radiological morphological manifestations of AVN are the result of a long-term developmental process, which necessitates continued observations to full skeletal maturity. Therefore, our results may not reflect the true incidence of AVN. To address this issue, we also analyzed another predictive indicator of AVN—HWI. This indicator, first proposed by Eyre-Brooke [23] in 1936, can be used to predict femoral head necrosis based on the evaluation of the morphological development of the capital femoral epiphysis. Therefore, it provides an indication of the long-term changes in AVN [11]. Our results showed that both groups had similar HWI at the final follow-up, which also does not support the presence of a link between PFO and femoral head morphological development. Furthermore, although the patients of this study were followed up for more than 2 years, the manifestations of the most common type of AVN (type II) usually appear at the age of 9 years. Therefore, our follow-up results do not provide an accurate evaluation of AVN incidence in the patients. In a previous study, we found that the Alsberg angle can be used as a predictive indicator of type II AVN [10]. However, in this study, the Alsberg angle at the final follow-up was not significantly different between the two groups, which also is not in favor of a relationship between AVN occurrence and PFO.

**Figure 2:**
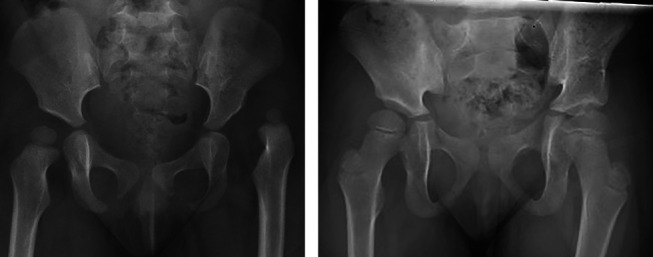
Postoperative development of the capital femoral epiphysis in the left hip in a female DDH patient who underwent OR and DA combined with PFO at an operative age of 17 months. (a): Preoperative AP pelvic radiograph showing a smaller capital femoral epiphysis on the left side; (b): 40-month postoperative AP pelvic radiograph showing a larger maximum transverse diameter of the capital femoral epiphysis on the left side (*r* = 1.16).

**Figure 3:**
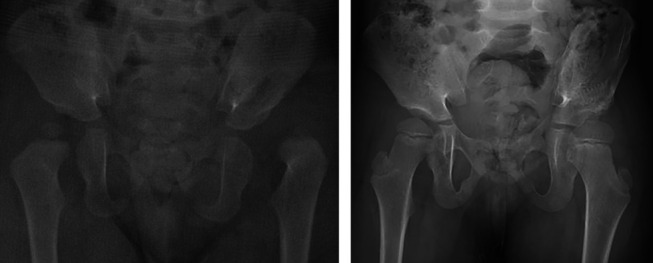
Postoperative development of the capital femoral epiphysis in the left hip in a female DDH patient who underwent OR and DA without PFO at an operative age of 19 months. (c): Preoperative AP pelvic radiograph showing a smaller capital femoral epiphysis on the left side; (d): 39-month postoperative AP pelvic radiograph showing a larger maximum transverse diameter of the capital femoral epiphysis on the left side (*r* = 1.42).

The *r* values of both groups at the final follow-up were >1.00, indicating that the maximum transverse diameter of the capital femoral epiphysis on the affected side was larger than that of the healthy side in the long term (Figures 2 and 3). Such a phenomenon is regarded as a type of AVN by Salter’s criteria but not classified as AVN according to the Kalamchi–MacEwen classification system. In our opinion, this result indicates the occurrence of post-reduction developmental disorders of the epiphysis rather than AVN. Studies have shown that femoral head enlargement of more than 15% can lead to adverse consequences such as poor acetabular coverage, acetabular sclerosis, and degenerative arthritis [24]. Therefore, we also compared the incidence of *r* values >15% at the final follow-up, but did not observe a significant difference between the two groups, which suggests that the occurrence of capital femoral epiphysis enlargement was not affected by PFO.

Proximal femoral shortening osteotomy can affect the long-term development of both lower limbs. Our results indicate a longer lower limb length on the affected side than on the healthy side at the final follow-up in unilateral DDH patients who underwent femoral shortening osteotomy. This phenomenon may be similar to the occurrence of post-fracture temporary over-lengthening of the femoral shaft in children. However, the same was observed in the non-PFO group, which has not been reported in previous literature and is of unknown cause. In a study including adult patients with untreated DDH, it was found that the femoral and tibia length were longer on the affected side than on the healthy side even before treatment [25]. Although we did not measure the pre-treatment lower limb lengths in DDH patients in this study, such a phenomenon cannot be ruled out. We surmise that postoperative over-lengthening of the affected side may be related to growth disturbances in the proximal femur after reduction. Although over-lengthening of the affected side at the final follow-up was more obvious in the non-PFO group, the difference between the two groups did not exceed 1 cm and was clinically insignificant.

This study has several limitations. First, the study was retrospective. Therefore, a certain degree of bias existed in case selection. Next, our overall sample size was small. Besides, as the included subjects had Tönnis type III and below DDH, the effect of PFO on Tönnis type IV DDH could not be evaluated. Lastly, the final AVN incidence could not be accurately determined due to the short follow-up time.

In conclusion, by comparing the post-reduction stability, acetabular remolding, proximal femoral morphological development, and AVN occurrence, we found no significant differences in the midterm outcomes of patients who underwent PFO and those who did not undergo PFO. Therefore, in walking-age children with Tönnis type III DDH or below, the midterm outcome is not affected by whether PFO is simultaneously performed during OR and DA.

## Data Availability

Data will be available upon request. Please contact the corresponding author.
